# Detection of genetic alterations in gastric cancer patients from Saudi Arabia using comparative genomic hybridization (CGH)

**DOI:** 10.1371/journal.pone.0202576

**Published:** 2018-09-13

**Authors:** Fehmida Bibi, Isse Ali, Muhammad Imran Naseer, Hussein Sheikh Ali Mohamoud, Muhammad Yasir, Sana Akhtar Alvi, Asif Ahmed Jiman-Fatani, Ali Sawan, Esam Ibraheem Azhar

**Affiliations:** 1 Special Infectious Agents Unit, King Fahd Medical Research Centre, King Abdulaziz University, Jeddah, Kingdom of Saudi Arabia; 2 Centre for Computational Intelligence (CCI), Faculty of Technology, De Montfort University, United Kingdom; 3 Center of Excellence in Genomic Medicine Research (CEGMR), King Abdulaziz University, Jeddah, Saudi Arabia; 4 Department of Clinical Genetics, St George’s University Hospitals NHS Foundation Trust, Cranmer Terrace London, United Kingdom; 5 Department of Medical Microbiology and Parasitology, Faculty of Medicine, King Abdulaziz University, Jeddah, Saudi Arabia; 6 Department of Anatomical Pathology, Faculty of Medicine, King Abdulaziz University, Jeddah, Saudi Arabia; 7 Department of Medical Laboratory Technology, Faculty of Applied Medical Sciences, King Abdulaziz University, Jeddah, Saudi Arabia; Chinese University of Hong Kong, HONG KONG

## Abstract

**Background:**

The present study was conducted to discover genetic imbalances such as DNA copy number variations (CNVs) associated with gastric cancer (GC) and to examine their association with different genes involved in the process of gastric carcinogenesis in Saudi population.

**Methods:**

Formalin-fixed paraffin-embedded (FFPE) tissues samples from 33 gastric cancer patients and 15 normal gastric samples were collected. Early and late stages GC samples were genotyped and CNVs were assessed by using Illumina HumanOmni1-Quad v.1.0 BeadChip.

**Results:**

Copy number gains were more frequent than losses throughout all GC samples compared to normal tissue samples. The mean number of the altered chromosome per case was 64 for gains and 40 for losses, and the median aberration length was 679115bp for gains and 375889bp for losses. We identified 7 high copy gain, 52 gains, 14 losses, 32 homozygous losses, and 10 copy neutral LOHs (loss of heterozygosities). Copy number gains were frequently detected at 1p36.32, 1q12, 1q22, 2p11.1, 4q23-q25, 5p12-p11, 6p21.33, 9q12-q21.11, 12q11-q12, 14q32.33, 16p13.3, 17p13.1, 17q25.3, 19q13.32, and losses at 1p36.23, 1p36.32, 1p32.1, 1q44, 3q25.2, 6p22.1, 6p21.33, 8p11.22, 10q22.1, 12p11.22, 14q32.12 and 16q24.2. We also identified 2 monosomy at chromosome 14 and 22, 52 partially trisomy and 22 whole chromosome 4 neutral loss of heterozygosities at 13q14.2-q21.33, 5p15.2-p15.1, 5q11.2-q13.2, 5q33.1-q34 and 3p14.2-q13.12. Furthermore, 11 gains and 2 losses at 1p36.32 were detected for 11 different GC samples and this region has not been reported before in other populations. Statistical analysis confirms significant association of *H*. *pylori* infection with T4 stage of GC as compare to control and other stages.

**Conclusions:**

We found that high frequency of copy number gains and losses at 1p36.23, 1p32.1, 1p36.32, 3q25.2, 6p21.33 and 16q24.2 may be common events in gastric cancer. While novel CNVs at 1p36.32 harbouring *PRDM16*, *TP73* and *TP73-AS1* genes showed 11 gains and 2 losses for 11 different GC cases and this region is not reported yet in Database of Genomic Variants may be specific to Saudi population.

## Background

Gastric cancer (GC) is one of the most silent killer in human, ranking as 9th common cancer in Saudi population while fourth common cancer in the world [1,2]. One of the main environmental factors for GC is *Helicobacter pylori* (*H*. *pylori*) infection that is initiated while taking rich salt, fat and sugary diet, and excessive tobacco usage particularly smoking, shisha pipe and poor sanitation. *H*. *pylori* cases are common in Saudi Arabia and other gulf countries [3], where people consume less fruits and vegetables but use excessive meat and unsaturated fat [4]. Gastric-cancer causes include both environmental and genetic factors. Recent studies focused on genetic factors as a risk for cancer where genetic alterations in several GC related studies have been reported to be involved in the development of GC [5]. Detection of GC at early stage is important for improvement of therapeutic measures use to reduce rate of morbidity. Significant numbers of studies have documented association of genomic alteration in cancer progression and development [6,7]. Several GC related studies have explored the association of genetic changes including mutation and duplication in GC progression [8]. In recent years, large-scale of individual genome have analysed to determine a broad range of genetic variants (CNV, SNP) to characterise genetic factor associate a specific phenotype. However, there are limitations in current technical DNA sequence strategy which lead on accuracy limitation of structural genomic variants.

In several types of cancer, DNA copy number variations (CNVs) are common. CNVs are rearrangements of DNA content either increase or decrease at certain region and are major source of genetic variations in humans. In GC, these CNVs are also an important indicator for risk and development of GC. It has been identified many of chromosomal aberrations including, monosomy, trisomy, gain, deletion, loss of heterozygosity (LOH), and neutral heterozygosity (NOH), indicate complexity of disease (cancer) progression. Recently, comparative genomic hybridization array (aCGH) has provided new insight into several genomic regions in GC patients that has gain and loss of DNA regions. Such as some regions showed gain of DNA including 1p, 6p, 3q, 7q, 8q, 17q and 20q and losses of DNA regions i.e 3p, 4q, 5q, 17p, 18q and 19p [9–13] in GC. These studies have documented that genetic instability may play an important role with the disease progression in GC.

Gastric cancer early detection is more important to prolong and improve life span where early stage of gastric cancer can be eliminated by surgical intervention. Finding causative biomarkers and novel diagnostic methods to detect the most common primary and secondary genes that causes the disease have always advantages for reducing disease risks. The Illumina Human Omni 2.5M genotyping SNP-array, the most effective method to detect whether a specific gene regulate as primary or secondary cause for disease. In GC, DNA copy number gains and losses are common and such analyses were not performed before in Saudi population from GC patients. Therefore, we designed a study using CGH array to provide an insight of genetic instabilities in GC (33 cases) formalin-fixed paraffin-embedded (FFPE) tissue specimens and normal control (15 cases), to identify chromosomal instabilities involved in initiation and progression of GC in Saudi population. In addition, we also study relationship of CNV profile and the transcriptome in GC.

## Materials and methods

### Clinical samples and ethical approval

Formalin-fixed paraffin-embedded (FFPE) tissues biopsy samples from 33 gastric cancer patients were collected from King Abdulaziz university (KAU) hospital Jeddah. Written consent forms were obtained from Gastric cancer ppatients undergoing a surgical procedure. Medical ethical committee of KAU, Jeddah Saudi Arabia has approved this study (Reference#174–15). Normal control gastric tissue samples were collected from 9 females and 6 males with an average age of 37.5 (range, 22–53) years. The GC patients consisted of 2 females and 6 males with an average age of 65 (range, 43–87) years from early stage and 4 females and 21 males with an average age of 54.5 (range, 27–82) years. All GC patients before surgery had no history of chemotherapy or radiotherapy. For GC samples, pathological staging was made according to tumor-node-metastasis (TNM) staging of the International Union against Cancer [14] and grading was performed according to the World Health Organization criteria [15]. In this study, 33 FFPE biopsy samples from gastric cancer patients were collected along with detailed clinical history from KAU hospital.

### DNA extraction and quality analysis

DNA from FFPE tissues was extracted using QIAamp® DNA FFPE Tissue kit according to manufacturer’s instructions. Using NanoDrop® ND-1000 UV-Vis Spectrophotometer, quality of DNA samples was checked. Using agarose gels electrophoresis, samples were electrophoresed and samples showing no smearing with intact genomic DNA selected for experiment. Quant-iT Picogreen (Invitrogen) quantification was used after diluted intact genomic DNA. Further prepared samples were hybridized to Infinium microarrays according to the manufacturer’s instructions.

### Sample genotyping using BeadChips and data analysis

Genome-wide SNP genotyping was performed using the Illumina HumanOmni1-Quad v.1.0 BeadChip (Illumina) according to manufactures instructions. To identify putative CNVs, the genotyped single nucleotide polymorphism (SNP) dataset of each sample was analyzed with the QuantiSNP algorithm v.2.2 using log2 R ratio (LRR) values and the B-allele frequency (BAF) values to generate CNV calls. Using Illumina’s Genome Studio software image intensities were extracted and intensity files were processed by using Genome Studio GT module 1.1.9. Each SNP is analyzed independently to cluster and identify genotypes.

Chromosomal aberrations were detected by making comparison of the normalized intensity of a GC sample to a reference samples. Genomic profiles were produced by using the Illumina Genome Viewer (IGV) and Chromosome Browser (ICV) of Illumina's BeadStudio2.0 software, to identify and annotate chromosomal alterations from SNP genotyping data.

### Gene-network analysis

To investigate associated genes in performing different molecular function and biological pathway, gene interaction analysis was performed for individual genes in which each gene network is displayed. Gene cards database (http://www.genecards.org) was used for searching gene-gene interaction network to identify gene-gene association, then we selected those that have a value of 0.7 or higher. Furthermore, UCSC genome browser and Broading software were used for searching gene variants in order to locate the number of genes that belong to a particular region (deletion or duplication) for each cancer cases. Furthermore, these set of genes were displayed by using interactive gene view software (http://software.broadinstitute.org/software/igv).

### Statistical analysis

Binary logistic regression was employed to assess risk association for those infected specifically with *H*. *pylor*i and gastric cancer using Minitab (17.0) and SPSS (22.0) statistical software packages. Chi-square was utilised for P-value calculation and information regarding GC stages is mentioned in tables (**Table 1 and S1 Table**). Odd ratio and probability were used to assess the risk association of different tumour stages e.g. T4 and metastases. Tumour types, TI-TIV, NI-NIII, Metastases (M), age and sex were used as predictors (**Table 1**). Odd ratio and confidence interval (CI) were calculated for each pair of tumour type and control cases by using chi-square at 95% significance level, and probability were estimated. In addition, normal probability plot was used to examine the model suitability for the data, and likelihood ratio test was used for evaluation and comparing tumour stages with multiple interaction assessment with deviance p-value.

**Table 1:** Clinopathological features of GC patients with normal control.

Sample ID	T	N	M	Age	Sex
**Early-stage**					
**14**	1	0	0	56	Female
**21**	2	0	0	78	Male
**22**	2	0	0	87	Male
**23**	2	0	0	52	Male
**24**	2	0	0	59	Female
**25**	2	0	0	72	Male
**26**	1	0	0	43	Male
**29**	2	0	0	62	Male
**Late-stage**					
**1**	3	2	0	53	Male
**4**	3	2	0	47	Male
**6**	4	1	1	59	Male
**8**	3	2	0	70	Female
**10**	4	3	1	55	Male
**12**	2	2	0	58	Male
**13**	4	3	1	18	Male
**15**	3	2	0	77	Male
**16**	3	2	0	48	Male
**20**	4	3	1	82	Female
**22**	4	1	0	72	Male
**28**	3	3	0	57	Male
**30**	2	2	0	55	Male
**31**	2	2	0	60	Male
**32**	2	2	0	45	Male
**33**	2	2	0	62	Male
**34**	3	3	1	71	Female
**35**	3	3	0	54	Male
**36**	3	3	0	65	Male
**37**	3	3	0	27	Male
**38**	3	3	0	48	Male
**39**	2	1	0	81	Female
**40**	2	1	0	58	Male
**41**	3	2	0	43	Male
**42**	3	2	0	61	male
**43**	2	2	0	43	Male
**44**	2	2	0	60	Male

## Results

### Detection of copy number variations in Gastric cancer using comparative genomic hybridization

**Fig 1:**
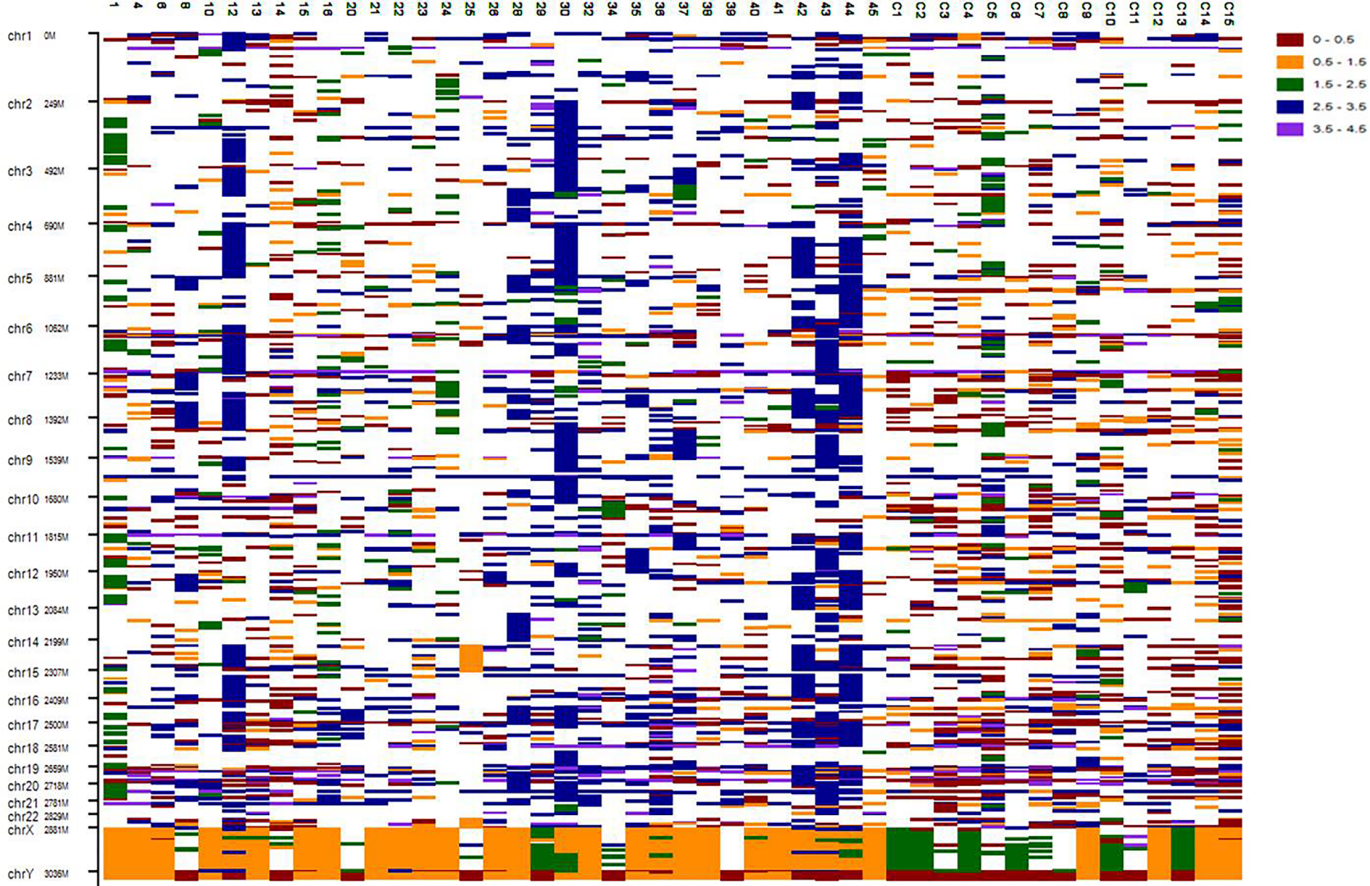
DNA copy number aberrations in gastric cancer and control samples. Homozygous loss (value, 0) were marked in brown, loss (value, 1) were marked with orange, copy neutral LOH (value, 2) were marked in green, gain were marked with blue (value, 3) and high copy gain were marked with purple (value, 4).

**Fig 2:**
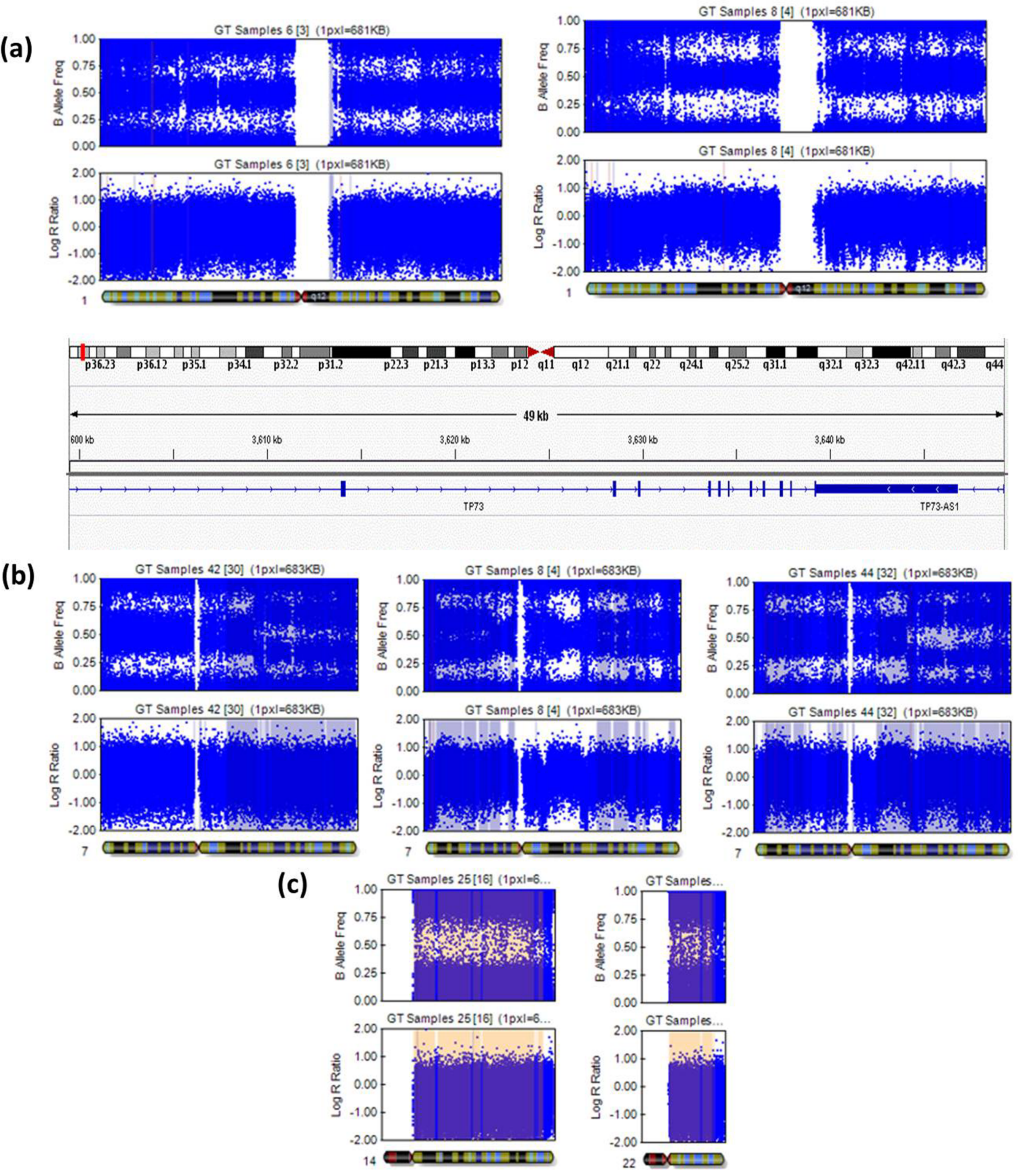
Shows loss and gain in chromosomes from different GC cases. **(a).** Homozygous loss in chromosome at 1p36.32 (3599473–3652626) for cases 6 and 8 of gastric-cancer late-stage tumor tissues. **(b)** Cases 8, 42 and 44 showed gain at 7p21.3-p36.3. **(c)** For GC sample 25, monosomy of chromosomes 14 and 22 in early stage gastric tumor tissue.

A total of 48 cases of GC were collected from King Abdulaziz University hospital and analyzed by CGH array. Ratio between male and female was 4:1 and 1:2 for GC and control samples. Patient’s median age is 53 (range, 18–87) and 38 (range, 22–53) for GC and control samples respectively. In 33 GC samples, twenty eight (80%) were with stages III-IV while seven samples (20%) were with stage I-II (**Table 1**). From 33 GC samples only 20 (60%) were positive for *H*. *Pylori* infection when tested by PCR [16]. We have targeted a large-scale of the whole chromosome to identify primary factors that cause gastric cancer, we employed high resolution array to characterise a common CNV in Saudi Arabia population. Using whole genomic array, and filtered out the common variants CNV which they don’t shared in gastric cancer cases were studied. This has used log-ratio three single base-pair which employed Illumina cnvPartition 3.2.1 algorithm. All CNVs reported in chromosomes were shown in **Fig 1**. We identified 7 high copy gain, 52 gains, 14 losses, 32 homozygous losses, and 10 copy neutral LOHs (loss of heterozygosities). We used threshold of 90% that is defined as CNVR copy number of variants region, and 100% of CNV overlap is defined as MCR (minimum common region). Whole genome sequence comparing the results and filtering of 30,000 regions is reduced to 85 regions. We also identified an additional CNV segments that have never been reported in the literature, where 13 (27%) out of 33 cases showed abnormalities on array analysis and the CNV regions are summarised in **Table 2**. We identified homozygous loss at region of 1p36.32, 1p32.1, 1q44, 3q25.2, 6p22.1, 6p21.33, 8p11.22, 10q22.1, 12p11.22, 14q32.12, 16q24.2, gain at 1p36.33, 1q12, 1q22, 2p11.1, 4q23-q25, 5p12-p11, 6p21.33, 9q12-q21.11, 12q11-q12, 14q32.33, 16p13.3, 17p13.1, 17q25.3, 19q13.32. We also identified 2 monosomy at chromosome 14 and 22, 52 partially trisomy and 22 whole chromosome 4 neutral loss of heterozygous at 13q14.2-q21.33, 5p15.2-p15.1, 5q11.2-q13.2, 5q33.1-q34 and 3p14.2-q13.12 and this will be interesting to find out the biological process for the genes of the affected regions. We also identified 11 gains and 2 losses at 1p36.32 for 11 different GC samples (9, 28, 4, 26, 3, 4, 15, 10, 13, 8, 6) and this region is not reported in Database of Genomic Variants (DGV). We identified 6 primary and 2 secondary causative genes found at 1p36.2 i.e *TP73* and TP*73-AS1* both showed homozygous loss in case 6 and 8 (**Fig 2A**). Comparing control and gastric cancer cases, we identified the most common genes and the chromosome positions that involved gastric-cancer progression including TP73-gene, *TP73-AS1* and CEP104 at chromosome 1q36.32 homozygous loss that is common in two cases (6 and 8), and also for the same chromosome at 1q36.1, gene *CDK18* found homozygous loss that is a primary cause of gastric-cancer. The same region 1q36.2 were also detected *PRDM16*, high copy gain for GC case 10, MiR4251 and *PRDM16* copy gain for case 28. At chromosome 1p36.32 showed copy number gains in GC samples from early stages while copy number losses were seen in late stages GC samples. In addition, region 1q36.33-1p36.32 copy gain were found 16 genes for case 30 suggesting these genes are primary cause gastric cancer. In current study also 11 gains have been seen for ten samples (6, 21, 22, 26, 34, 35, 36, 38, 41, 44) at chromosome 1q21.3-q22 harbouring *RAB13* and *MUC1* gene respectively. Case 12 and 44 have gain in same chromosome position at 7q22.1-q36.3. In addition, GC cases 12 and 44 found whole chromosome gain for the same position of chromosomes 5, 14 and 15 at the region of p15.33-q35.3, q11.1-q32.33, and q11.1-q26.3, respectively. We also found homozygous loss in case 1, 6, 10, 14, 24, 43 at chromosome 6p21.33. In addition, Cases 8, 42 and 44 found gain chromosome 7 at 7p21.11-p26.3, 7q22.3-q36.3 and 7q21.11-q36.3, respectively (**Fig 2B**). We found homozygous loss in 15 different GC cases (6, 12, 14, 22, 23, 25, 29, 30, 35, 36, 38, 41–44) at chromosome 1q12.3-1q13.2. For GC cases 8 and 44 gains were found at the same position of chromosome 8 at 8p21.11-q24.3, case 43 and 44 have the same position of chromosome 17 affected at 17q21.2-q22. There are other cases that share the affected position, 8 and 28 found gain at 5p15-p12, 8 and 12 share 8p23.3-p11.21, cases 12 and 30 found the same affected region at 3p26.3-p11.1, 20 and 30 gained at 16q11.2-q24.3, cases 37, 42 and 44 affected the same position at 11p15.5-p11.2, and 30 and 43 found affected common region at 11q11-q25. Monosomy can be seen in **Fig 2C** where GC case 25 was identified with monosomy on chromosomes 14 and 22. We have also investigated the ratio of copy number of gain and losses, number gains are found less frequently than the losses in generic region, suggesting copy number gains are less likely to be deleterious and therefore, less likely to incur a penalty in evolutionary selection.

**Table 2:** Summary of clinical data and abnormal array-data for gastric cancer of chromosome aberration.

Sample.ID and age	Gender	Stage in Carcinoma	Cancer-stages/pylori	Cytoband	Copy Neutral LOH	Start	Stop	Size	GeneSymbol
**Case25 (72)**	M	Early-stage	ES(+)	monosomy 14, monosomy 22						
**Case6 (59)**	M	Late-stage	ES(+)	** **1p36.32(3601798–3652626)		**3601798**	**3652626**	**50828**	**TP73,TP73-AS1**	
**Case8 (70)**	F	Late-stage	LS(+)	5p15.33-p12, 7p22.3-q1 1.22, 7q22.3-q36.3, 8p23.3-p11.21, 12 p13.2-q15, 19q13.32-q13.43						
**Case10 (55)**	M	Late-stage	LS(+)	12p13.33-p11.1, 13q14.2-q21.33, 18q21.2-q23, 20p13-p11.1, 21q21.1-q21.2,	13q14.2-q21.33					
**Case12 (58)**	M	Late-stage	LS(+)	trisomy 4, trisomy5, trisomy 6,trisomy 14,trisomy 15, trisomy 16, 1p36.33-p32.3, 2q12.1-q23.3, 3p26.3-p11.1, 7 q22.1-q36.3, 8p23.3-p11.21, 8 p24.3-q21.11, 17q21.33-q22						
**Case20 (82)**	F	Late-stage	LS(+)	16q11.2-q24.3, 17q21.2-q22						
**Case28 (57)**	M	Late-stage	LS(-)	trisomy 19,5p15.33-p12, 6p25.3-p11.1, 7q21.11-q22.1, 9q31.31-q34,13q31.31-q34, 16q32-q34.3, 18q13.1-q34, 20p13-p11.23						
**Case30 (55)**	M	Late-stage	LS(+)	trisomy 2, trisomy 4, trisomy 9,3p26.3-p11.1, 6 p25.3-q16.3, 8p21.3-q24.3,11q22.3-q25, 16q11.2-q24.3, 17p13.3-p11.2,18q11.2-q23, 20p13-q13.33	5p15.2-p15.1					
**Case35 (54)**	M	Late-stage	LS(-)	1p36.33-q24.3, 11q11-q25						
**Case37 (27)**	M	Late-stage	LS(-)	3p26.3-p14.2, 8q11.1-q24.3, 11p15.5-p11.12	3p14.2-q13.12					
**Case42 (61)**	M	Late-stage	LS(+)	trisomy 5, trisomy 12, trisomy 13,trisomy15, trisomy 17,2q11-q35.2, 4q21.11-q36.3, 7p24.1-p11.2,9q31.1-q34.3, 11p15.5-p11.12						
**Case43 (43)**	M	Late-stage	LS(+)	trisomy 6,8p23.3-p11.1, 8q21.11-q24.3, 9p24.3-p11.2,11q11-q25, 13q11-q13.3, 17q11.2-q25.3						
**Case44 (60)**	M	Late-stage	LS(+)	trisomy 5, trisomy 12, trisomy 14,trisomy 15, trisomy 17, trisomy 19,2q11-q35.2, 4q21.11-q36.3, 7p24.1-p11.2,9q31.1-q34.3, 11p15.5-p11.12						

(Number) represent age of patient, M = male, F = female, LS = late stage, ES = early stage and (+,-) represent CNV Gain and CNV loss respectively.

### Copy number variations and candidate genes

**Fig 3:**
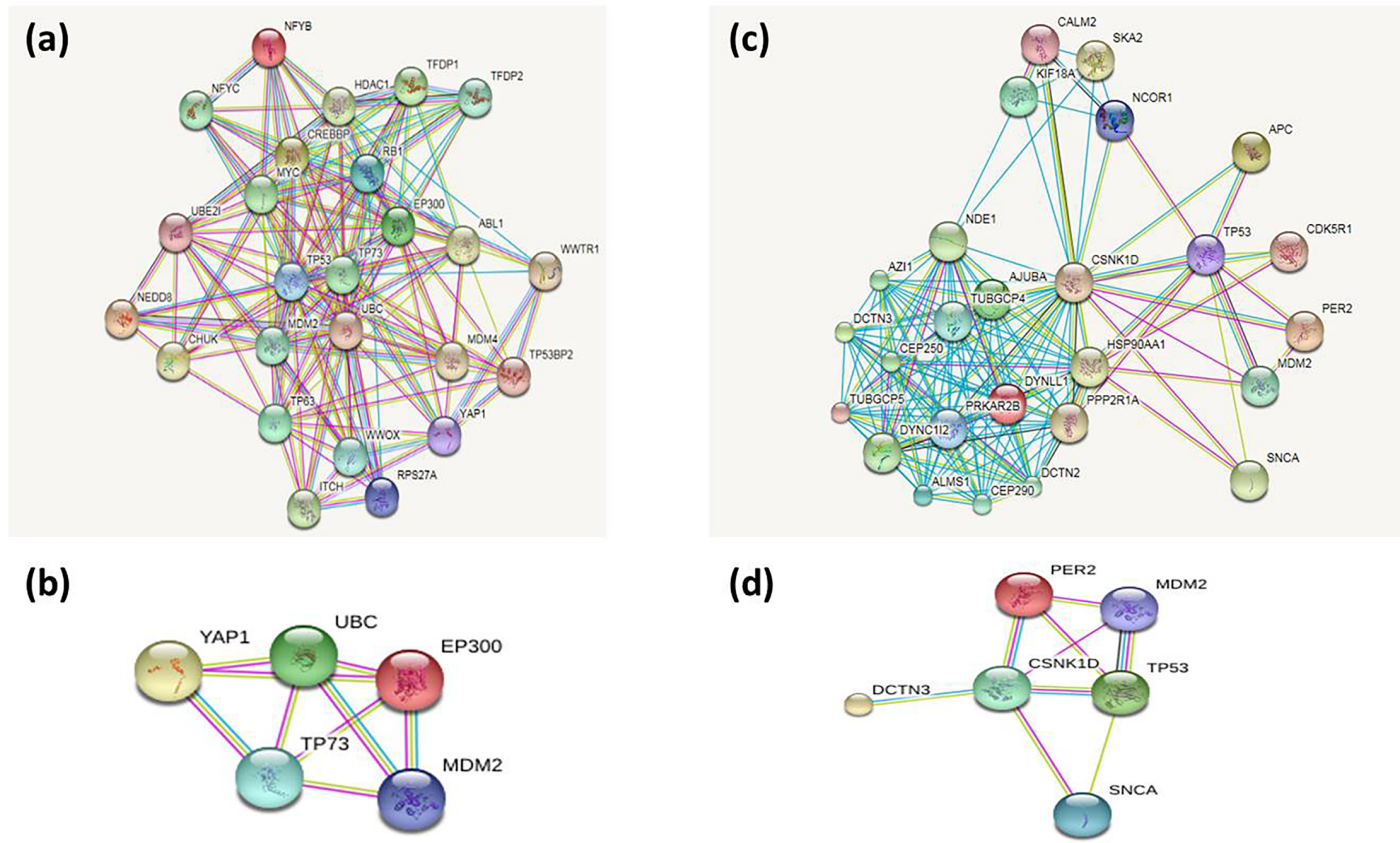
Gene-gene interaction indifferent GC tissue samples. **(a).** Edges represents jointly contribute gene-gene interaction, illustrates TP73 gene-gene network upper show the first 25 genes that interact with TP73 gene. **(b)** down figure show four genes that interact with TP73. **(c)** PER2 gene-gene network upper 25 genes that interact with PER2 gene. **(d)** down figure show four genes that interact with PER2. Light blue lines represent annotated interaction genes, red represents experiment determined genes, and yellow lines represent text-mined gene-interaction.

**Fig 4:**
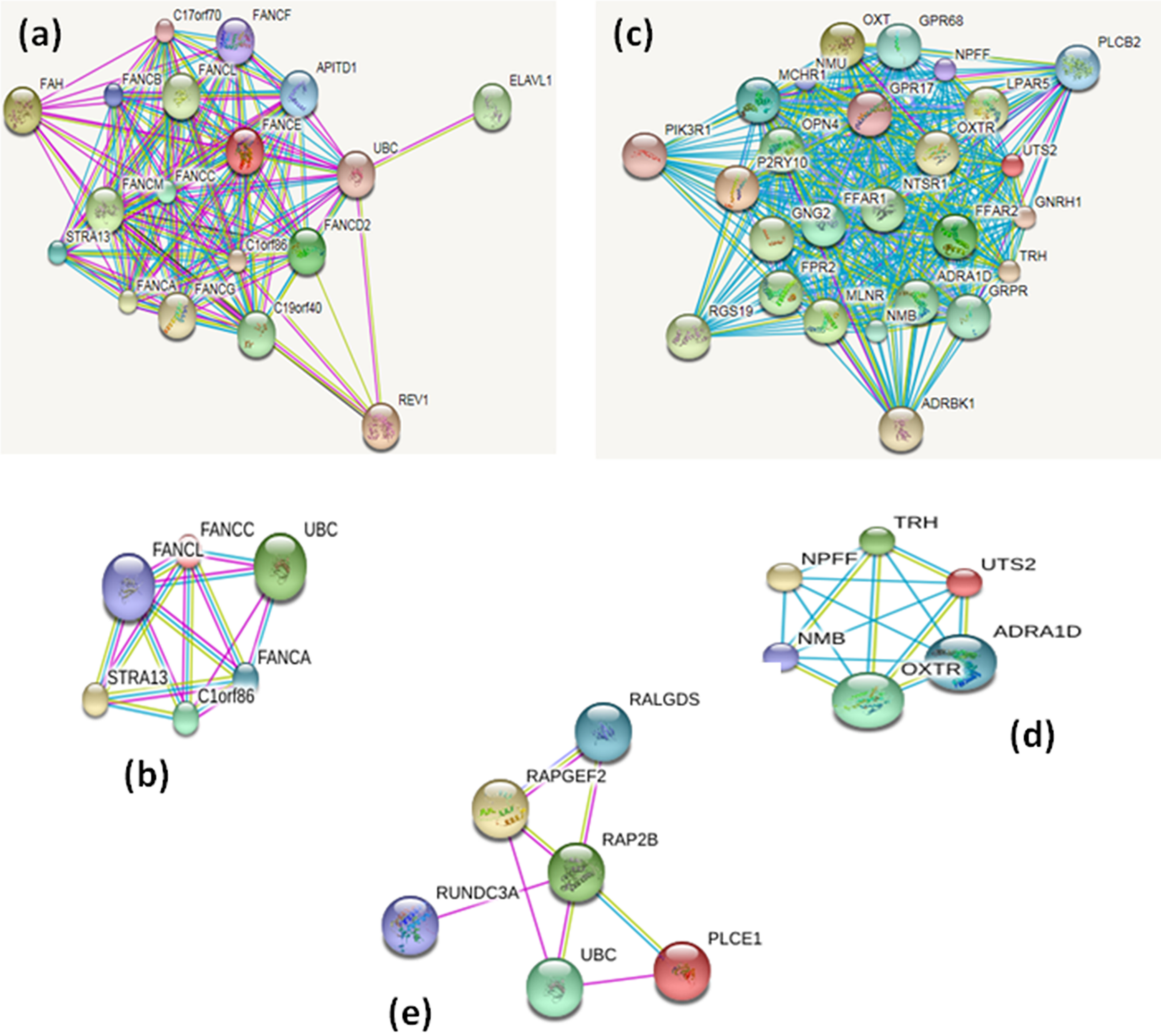
Shows signalling of different gene network in Gc samples. **(a)** Edges represents jointly contribute gene-gene interaction, illustrates upper show first 25 genes that interact with C1orf86 gene and gene-gene network. **(b)** down figure show four genes that interact with C1orf86. **(c)** the upper figure show the first 25 genes that interact with UTS2 gene. **(d)** UTS2 gene-gene network down figure show four genes that interact with UTS2. Light blue lines represent annotated interaction genes, red represents experiment determined genes, and black lines represent co-expression gene-interaction.

We have also investigated gene network in order to identify their gene-gene interaction. For example, *PER2* showed connection with 25 different genes in gene-gene interaction. Where *CSNK1D*, *TP53* and *MDM2* show direct connection to *PER2* which suggest that these may have protein interaction and other two genes (*SNCA* and *DCTN3*) have indirect connection. *MDM2* is a proto-oncogene regulates *TP53*, these genes may contribute to the gastric cancer through gene network interaction (**Fig 3A–3D**). However, there is a little understanding how *TP53* transcriptionally regulate *PER2* to cause gastric cancer. Amplification at 1p36.32 was higher and candidate genes for this chromosome are *TP73* and *TP73-AS*. *TP73* belong to family member of transcription factor TP53 which regulates cancer pathways. At 1p36.33-p36.32, thirteen genes were identified the first three genes are *C1orf86*, *FAM213B*, and *HES5*. C1orf86 at 1p36.23 (7809383–7905274) were identified four genes gain e.g *CAMTA1*, *PER2*, *UTS2* and *VAMP3*. UTS2 involves signalling pathways and *UTS2* mutation is associated with different types of cancer (**Fig 4A–4D)**. *PER2* regulates through interaction of various cellular signalling including, *TP53* and *CSNK1D* suggesting *PER2* gene regulates indirect for these genes which possibility they cause gastric-cancer. In addition, homozygous loss at 3q25.2 (152818434–152857465) encode RAP2B gene, which interacts *RAPGEF2*, *RALGDS*, and *RUNDC3A* (**Fig 4E)**. The interested gene regions are displayed, such as copy number gene, exonic region and overlapping features were zoomed to display all features (**S1, S2, S3 and S6 Figs**).

### Binary logistic regression analysis

**Fig 5:**
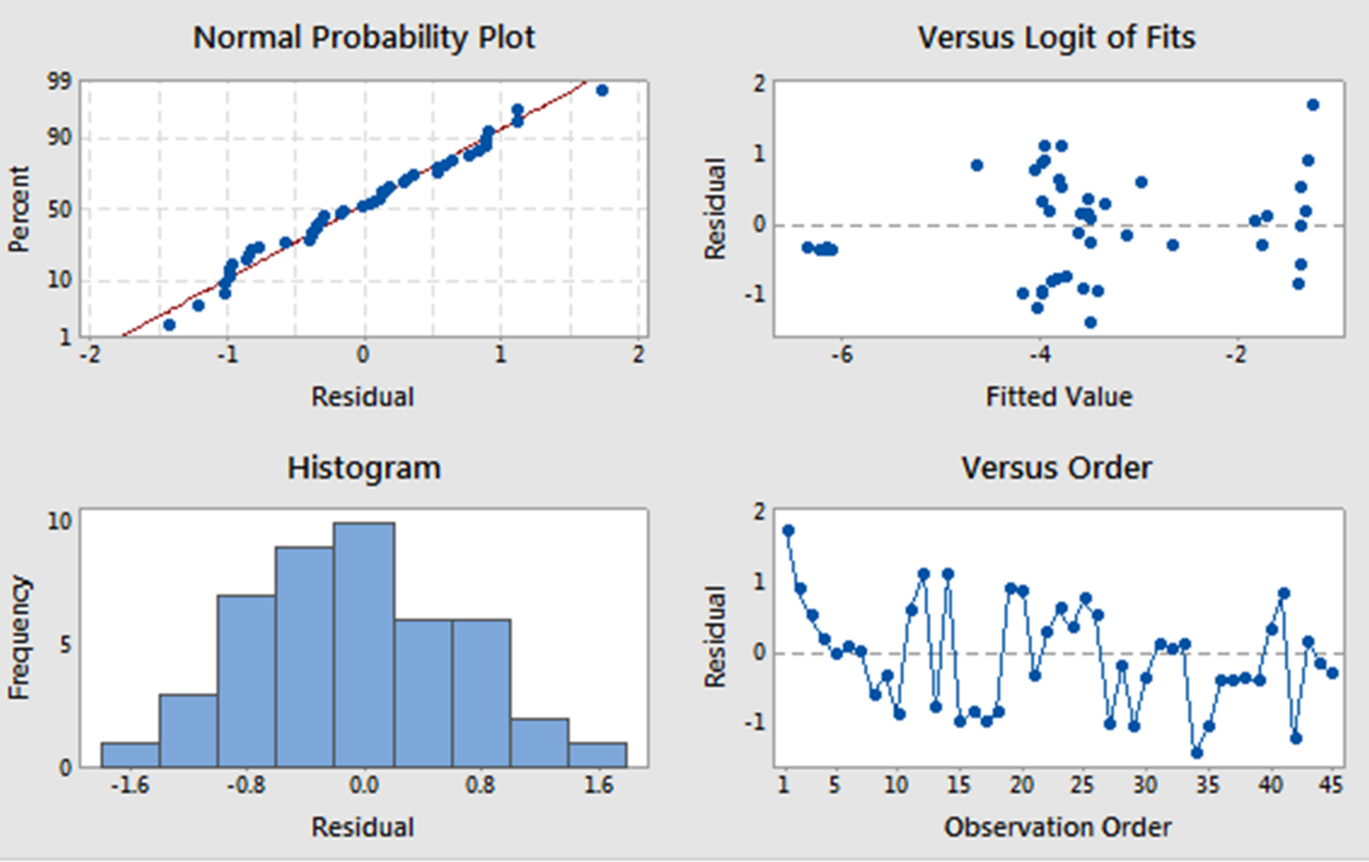
Deviance residual plot for H. pylori versus T, N, M, sex and age. (a) Normal probability plot. (b and d) residuals show vary randomly scattering with a constant pattern.(c) histogram show symmetrical distribution; therefore, the fitted regression model is visually well suitable for the data. Note, Chi-square test at 95% significant level with p-value = 0.000.

We have compared different cancer stages against control and among them to estimate the probability that the patient develops higher stages when infected with *H*. *pylori*, and the possibility associated with metastases risks. The method used builds binary logistic regression, which successfully estimated the risk association of *H*. *pylori* infection in different tumour stages with P-value (0.000) less than 0.05. The fitted model illustrated in **Fig 5** which confirms error follows a normal probability distribution; therefore, the fitted regression model is visually well suitable for the data, and P-value provided by the deviance at 95% significant level in Chi-square distribution was 0.91. This is indeed greater than 0.05 suggesting our model fits the data adequately. Tumour at stage IV (T4) was significantly associated with *H*. *pylori* infection compared to the control and type (I-III) cases (p<0.05). The Odd ratio for T4 was 2.68 (95% Confidential interval; 95% CI; 02557–28.0756). In addition, metastases cases were also significantly associated with *H*. *pylori* infection with p<0.05. The odds ratio value was 4.05 (95% Confidential interval; 95% CI; 0.3227–50.9119), this suggests an increased risk of metastases, and GC cases which were *H*. *pylori* positive had a probability value of 0.8 compared to the unaffected cases (*H*. *pylori* negative cases). Furthermore, there is no significant risk associated between age and sex, and *H*. *pylori* infection (**S2 Table**). Although, when all predictors (categories) were combined together, it enhances statistical power with p-value (P<0.001). The most frequency genes are TP73 (both alleles loss) and PRDM16 (high copy gain) both are associated with late cancer stages which may cause uncontrolled cell division due to these genes changes. Moreover, all primary cause genes were validated in a curetted catalogue of human genomic structural variation database (http://dgv.tcag.ca/gb2/gbrowse/dgv2_hg38/). We found that CDK18 was one in sixteen thousand cases. In addition, logistic regression results show that T4 have odd ratio value of 2.68 (**Table 3 and S2 Fig**), which is 2.68 times more chance for cancer than those who were *H*. *pylori* positive compared type stages (I-III), and 4 times the metastases cases compared to *H*. *pylori* negative cases in **Table 3**. *H*. *pylori* infection may be one of the important factors for gastric cancer for diverse cancer stages.

**Table 3:** Logistic regression analysis in GC cases.

Categorised predictors	Parameter estimate	Standard error	Estimated risk (odd ratio)	95% CI	P-value
**Age**	0.0066	0.07	1.01	0.97–1.04	0.700
**T1**	-1.78	1.11	0.17	0.05–8.03	0.107
**T2**	-2.24	0.99	0.63	0.05–8.03	0.025
**T3**	-2.66	1.17	0.66	0.20–2.13	0.023
**T4**	-1.67	1.59	2.68	0.26–28.08	0.0293
**N1**	-0.72	1.10	0.49	0.06–4.17	0.510
**N2**	0.42	0.84	3.12	0.519–18.77	0.0620
**N3**	-1.83	1.33	0.33	0.05–2.33	0.169
**M**	1.4	1.29	4.05	0.32–50.91	0.0278
**Sex**	-0.51	0.50	0.60	0.23–1.60	0.309

## Discussion

In this study, we employed integrated whole genome techniques with high array-resolution to identify a common CNV’s in gastric cancer cases vs control samples from Saudi patients. We also analyzed different gene pathways to find oncogenes and tumor suppressor genes in GC patients. The ratio of total gain and loss counts of CNVs were reported 72.6% of all variants which have a copy number L2 per diploid [17,18] reported 20,099 CNV in Asian population, where 670 were reported which covered 11.31kmb of total DNA sequence. Chromosomal imbalances are major factor of genetic changes in GC. Previous studies have highlighted chromosomal instabilities in GC tumor samples analyzed by aCGH [19–21].

Previous studies have documented role of tumor-suppressor genes associated with gastric cancer exist on different loci at chromosomal arms lp, lq, 5q, 7q, 12q 17p and 18q [22,23]. We identified 4 homozygous loss at region of 1p and loss of allele at chromosome 1p is reported to have association with different human malignancies such as hepatic, gastric and thyroid carcinomas [23–25]. Chromosome 1p loss is also documented in brain tumor and two different regions 1p36 and 1p32–p35 contain loci responsible for development of tumor [26]. One of the most likely candidate genes in this region is *TP73* which share structural and functional homology with *TP53*. *TP53* is a tumor suppressor gene in neuroblastoma and other cancers and function to delay the cell cycle in case of DNA damage until it get repaired. *TP73* was first reported as a candidate suppressor gene localized at chromosome 1p36.3 in neuroblastoma [27]. Our data has also revealed for the same chromosome at 1q36.1 found homozygous loss that is consistent with previous study [22]. *TP73* was found high expression in cervical cancer cases [28], however it is not reported for gastric cancer in literature. In this study we employed SNP-array to identify indels particularly primary and secondary causes genes that regulates gastric cancer. Few studies have been done so far related to GC in Saudi Arabia. In our recent study we have reported expression of different miRNAs, where miR-200c-3p was potential biomarker in both early and late stage GC cases among others in Saudi population [4].

*H*. *Pylori* infection develops and progress steadily state of steps from inflammation to atrophy, metaplasia and dysplasia. Chronic gastritis after *H*. *pylori* infection activates inflammatory signaling pathways that may initiate gastric tumorigenesis [29]. In chronic infection with *H*. *pylori* genes of epithelial cells of stomach mutated and resulted in inhibition of apoptosis and increased cell proliferation. The gastric epithelium cells after the presence of mucosal pathogens express different cell surface receptors and activate inflammatory pathways [30]. Mucin (MUC) family includes proteins that play important roles in protecting epithelial cells and pathogens, and help in renewal and differentiation of epithelial cells. MUC1 is an oncogene in cancer cells while in normal cells plays a role to protect gastric epithelial cells from pathogens that initiate inflammation and carcinogenesis. In Chinese and Japanese populations GC patients chromosome 1q22 is a susceptibility locus harboring MUC1 gene [31]. In our previous study, 58% GC samples were positive for *H*. *Pylori* infection where 129 genes were highly expressed while 953 down-regulated and 33 miRNA highly expressed in both early and late of gastric cancer tissue [4]. In our study gains also have been seen at chromosome 1q21.3 and 1q22 harbouring *MUC1* and RAB13 gene respectively.

Furthermore, homozygous loss at cytoband 1q32.1 showed involvement of gene *CDK18*. Cyclin dependent kinases (CDKs), belong to a family of protein kinases which play critical function in regulating different cellular mechanisms as well as transcription. Dysregulation of CDKs results in imbalance in apoptosis and proliferation which is a hallmark of a cancer. Loss of *CDK18* may lead to dysregulation of cell-cycle check-point increasing genomic instability and development of gastric cancer [32]. Another gene that was related to GC identified in this study is PR domain containing 16 (*PRDM16*) which is function as a zinc finger transcription factor regulates chromatin function and different transcription factors. It also plays an important role in adipose tissue differentiation and described as an oncoprotein in different types of cancer including GC [33]. Furthermore a loss of PRDM16 was found a severity of hematopoietic stem cell activity [34]. The same researcher reported rearrangement of *PRDM16* and *EVI1* genes showed poor prognosis in AML cancer-cases, suggesting that *PRDM16* involves in development of gastric cancer. *RAP2B* is a RAS oncogene family member gene, reported to up regulate in various human cancers and involves in the progression of cancer. *RAP2B* loss may contribute dysregulation of RAS pathway which may cause genomic instability leading target interaction genes and gastric cancer progression [35].

We have seen higher frequency of chromosomal gains on 20q and p that is consistent with previous studies [11,36]. Gains at 20q play an important role in cell immortalization and pathogenesis [37]. Candidate gene for this region is FRG1B (FSHD region gene 1 family, member B) is a mutation driver gene play a role in progression of thyroid cancer [38]. Copy number gains reported in this study at p36.32-p36.33 where *C1orf86* is located. *C1orf86* is associated with genomic stability regulates cell survival followed by DNA damage and found its dysregulation linked to cancer predisposition syndrome leading genomic instability [39]. *FAM213B* associates with cell metabolism process through fatty acid biosynthesis [40]. Suggesting *FAM213B* contributes gastric cancer. *HES5* show direct interaction with notch signalling pathway and its dysregulation may lead to transcriptional disruption which could increase genomic instability and further cancer progression. *HES5* mRNA was found high expression in brain tumor tissue and in fetal heart [41], but it has not reported for gastric cancer. Genetic variability has been revealed in different stages of cancer between early and late stages. We have noticed that 1p36.32 showed copy number gains in GC samples from early stages while copy number losses were seen in late stages GC samples. No other significant correlation has been found for other gains or losses at different stages. Copy number gains and losses were frequently detected at 1p36.32, 3q25.21, 6p21.33 and 16q24.2. Statistical analysis also revealed that *H*. *pylori* infection was significantly associated with T4 stage of GC as compare to stage T1-3 and control. Here we have also have limitation due to small number of samples which may affect our results. More number of samples are needed in future to validate these results.

## Conclusions

In conclusion, the present study revealed that DNA copy number gains at 1p36.32, 2p11.1, 4q23-q25, 5p12-p11, 6p21.33, 9q12-q21.11, 12q11-q12, 14q32.33, 16p13.3, 17p13.1, 17q25.3, 19q13.32, and losses at 1p36.23, 1p36.32, 1p32.1, 3q25.2, 6p21.33, 8p11.22, and 16q24.2 may be common in GC. However, loss at 1p36.32 may be specific to Saudi population. These results establish the risk association between the tumour stages and *H*. *pylori* infection for further elucidation, gene expression is still being favoured as the method of biomarker detection, where the priority aim is to identify specific gene variants for Saudi Arabian population. The results open a great opportunity to the researchers; however, gene-expression profiling is needed only on the high copy gain regions for CNV, in fact we believe these primarily results can be useful for the genetic variant for Saudi Arabia population. In addition, we have identified some CNVs that have not been reported in previous reports. Our results identified potential genes involved in pathogenesis and progression of GC may be used as potential biomarkers for GC for although need further studies to be performed on larger samples. Taken together, these results play important role in understanding of gastric carcinogenesis in GC patients from Saudi Arabia.

## Supporting information

S1 Table Showing information regarding normal control and GC patients data used for binary logistic regression analysis. (DOCX)

S2 Table Estimation of risk association of H. pylori infection in different tumour stages using binary logistic regression analysis. (DOCX)

S1 Fig Visual representation of genes (CAMTA1, VAMP3, PER3 and UTS2) at chromosome 1(Chr1:7607329–8107328) used by the UCSC Genome Browser (http://genome-euro.ucsc.edu/cgi-bin/hgTrack). For example, gene regions are showed as compact blocks linked by thin-lines indicating introns. Overlapping features, such as multiple isoforms for a gene, were zoomed to display all features.
(TIF)

S2 Fig Shows gene copy number regions as solid blocks joined by thin lines which represent introns. These genes (CAMTA1, VAMP3, PER3 and UTS2) from chromosome 1(Chr1:7792484–8113343) copy gain region. Colour codes indicate, with red-dots are significant cis-eQTLS for the queried gene which less than at FDR<0.5 and grey-dots are significant cis-eQTLS for all other SNP-gene within the genomic region. This was used for Broading software (http://software.broadinstitute.org/software/igv/home).
(TIF)

S3 Fig Shows gene copy number regions as solid blocks joined by thin lines which represent introns. These genes (C1orf86, FAM213B, and HES5) from chromosome 1(Chr1:1819996–3028345) are copy gain genes. Colour codes with red-dots are significant cis-eQTLS for the queried gene which less than at FDR<0.5 and grey-dots are significant cis-eQTLS for all other SNP-genes within the genomic region. This was used for Broading software (http://software.broadinstitute.org/software/igv/home).
(TIF)

S4 Fig Figure shows PRDM16 gene pathways of white and brown adipocytes transcription factors and nuclear regulators directing the process of the PRDM16 by using Genecards database (http://www.genecards.org/cgi-bin/carddisp.pl?gene=PRDM16). (TIF)

S5 Fig This boxplot shows the expression values in transmission per million calculated from gene model with isoform collapsed as a single gene with no other normalisation were applied. Median, the first and the third quartile were calculated. This was used for Broading software (http://software.broadinstitute.org/software/igv/home).
(TIF)

S6 Fig Gene copy number regions as solid blocks joined by thin lines which represent introns. These genes (TP73 and TP73-AS1) from chromosome 1(Chr1:3572898–3679203) LOH region. Colour codes, with red-dots are significant cis-eQTLS for the queried gene which less than at FDR<0.5 and grey- dots are significant cis-eQTLS for all other SNP-genes within the genomic region.This was also used for Broading software (http://software.broadinstitute.org/software/igv/home).
(TIF)
