# Supplementary material for: Detection of genetic alterations in gastric cancer patients from Saudi Arabia using comparative genomic hybridization (CGH)
Source: PLoS One. 2018 Sep 13;13(9):e0202576. doi: 10.1371/journal.pone.0202576 (PMC6136709; doi:10.1371/journal.pone.0202576)
Supplement: S1 Table — (DOCX) [file pone.0202576.s001.docx]

| **Sample** | **Number** | **Sex** | **Stage** |
| --- | --- | --- | --- |
| **Normal Gastric tissue samples** | 9 | Female | Normal |
|  | 6 | Male | Normal |
| **Gastric cancer patient** | 2 | Female | Early-stage |
|  | 6 | Male | Early-stage |
|  | 4 | Female | Late-stage |
|  | 22 | Male | Late-stage |

**S1Table**
