# Supplementary material for: Detection of genetic alterations in gastric cancer patients from Saudi Arabia using comparative genomic hybridization (CGH)
Source: PLoS One. 2018 Sep 13;13(9):e0202576. doi: 10.1371/journal.pone.0202576 (PMC6136709; doi:10.1371/journal.pone.0202576)
Supplement: S2 Table — (DOCX) [file pone.0202576.s002.docx]

| **Coefficients** |
| --- |
| **Term Coef SE Coef 95% CI Z-Value P-Value VIF** |
| **Constant -1.562 0.762 ( -3.055, -0.068) -2.05 0.040** |
| **age 0.0066 0.0172 (-0.0271, 0.0403) 0.38 0.700 2.07** |
| **t** |
| **1 -1.78 1.11 ( -3.95, 0.38) -1.61 0.107 1.14** |
| **2 -2.242 0.999 ( -4.200, -0.283) -2.24 0.025 6.29** |
| **3 -2.66 1.17 ( -4.95, -0.36) -2.27 0.023 5.53** |
| **4 -1.67 1.59 ( -4.79, 1.45) -1.05 0.294 8.37** |
| **n** |
| **1 -0.72 1.10 ( -2.87, 1.43) -0.66 0.510 3.11** |
| **2 0.416 0.839 ( -1.229, 2.060) 0.50 0.620 4.61** |
| **3 -1.83 1.33 ( -4.44, 0.78) -1.38 0.169 4.62** |
| **m** |
| **1 1.40 1.29 ( -1.13, 3.93) 1.08 0.278 5.51** |
| **gender** |
| **2 -0.508 0.499 ( -1.486, 0.470) -1.02 0.309 1.55** |
|  |

**Table S2. (regression coefficients)**
