# Supplementary figures and images for: Detection of genetic alterations in gastric cancer patients from Saudi Arabia using comparative genomic hybridization (CGH)
Source: PLoS One. 2018 Sep 13;13(9):e0202576. doi: 10.1371/journal.pone.0202576 (PMC6136709; doi:10.1371/journal.pone.0202576)

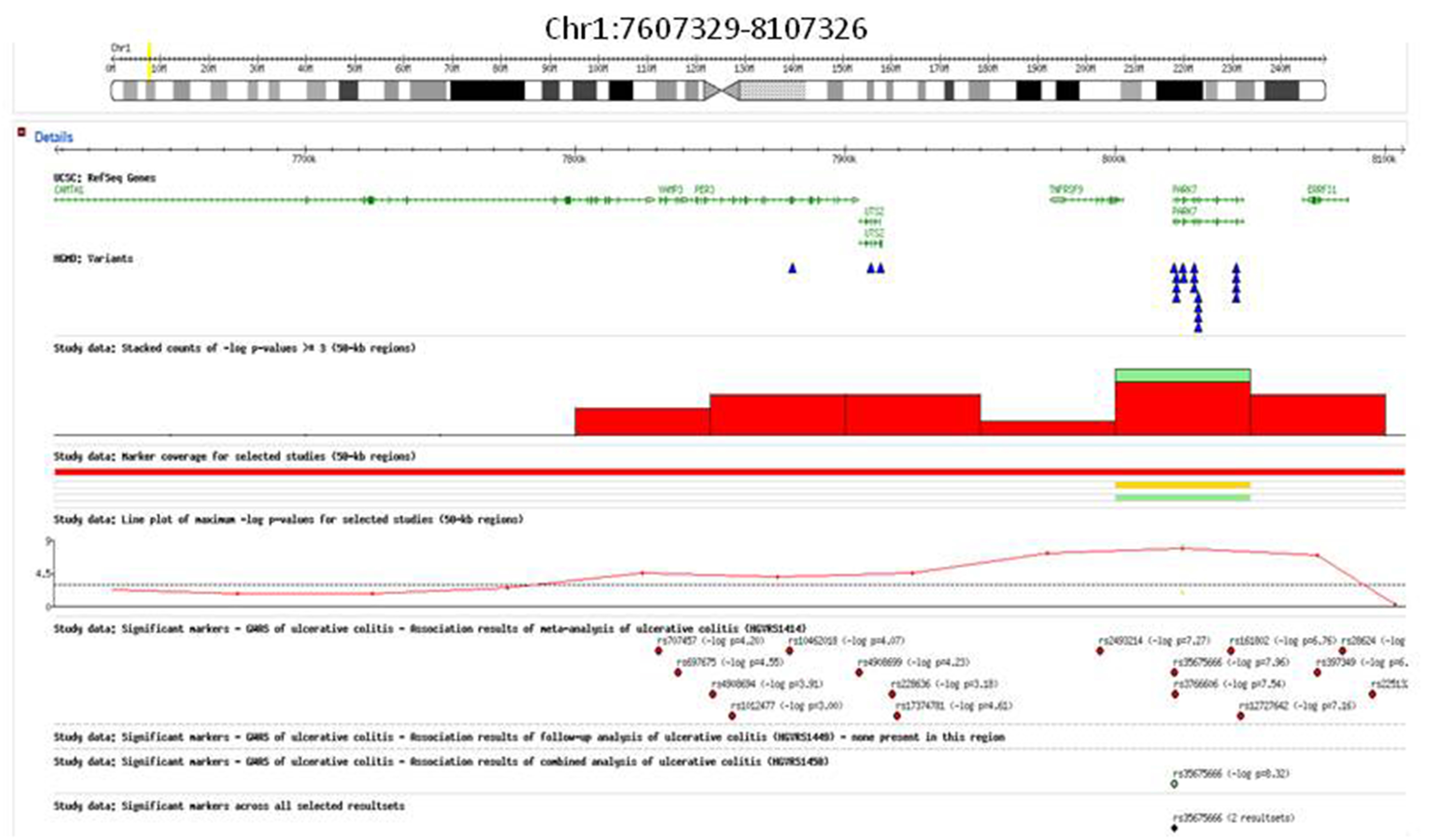

Supplement: S1 Fig — For example, gene regions are showed as compact blocks linked by thin-lines indicating introns. Overlapping features, such as multiple isoforms for a gene, were zoomed to display all features. (TIF) [file pone.0202576.s003.tif]

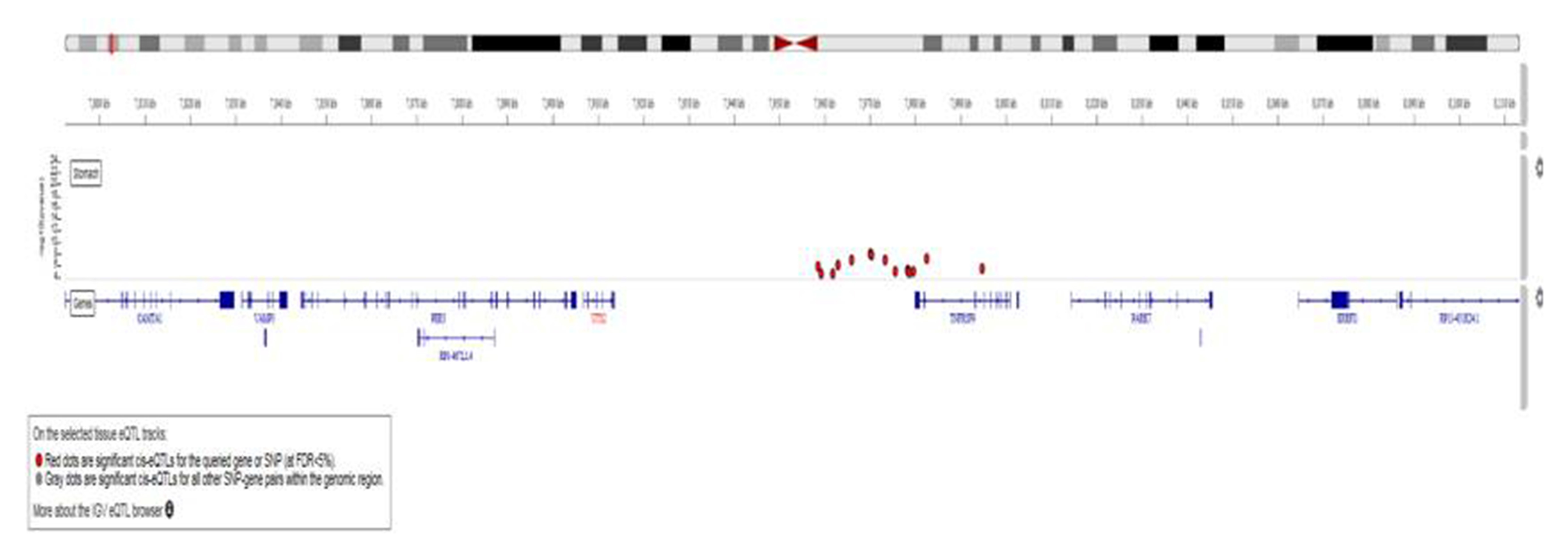

Supplement: S2 Fig — These genes (CAMTA1, VAMP3, PER3 and UTS2) from chromosome 1(Chr1:7792484–8113343) copy gain region. Colour codes indicate, with red-dots are significant cis-eQTLS for the queried gene which less than at FDR<0.5 and grey-dots are significant cis-eQTLS for all other SNP-gene within the genomic region. This was used for Broading software (http://software.broadinstitute.org/software/igv/home). (TIF) [file pone.0202576.s004.tif]

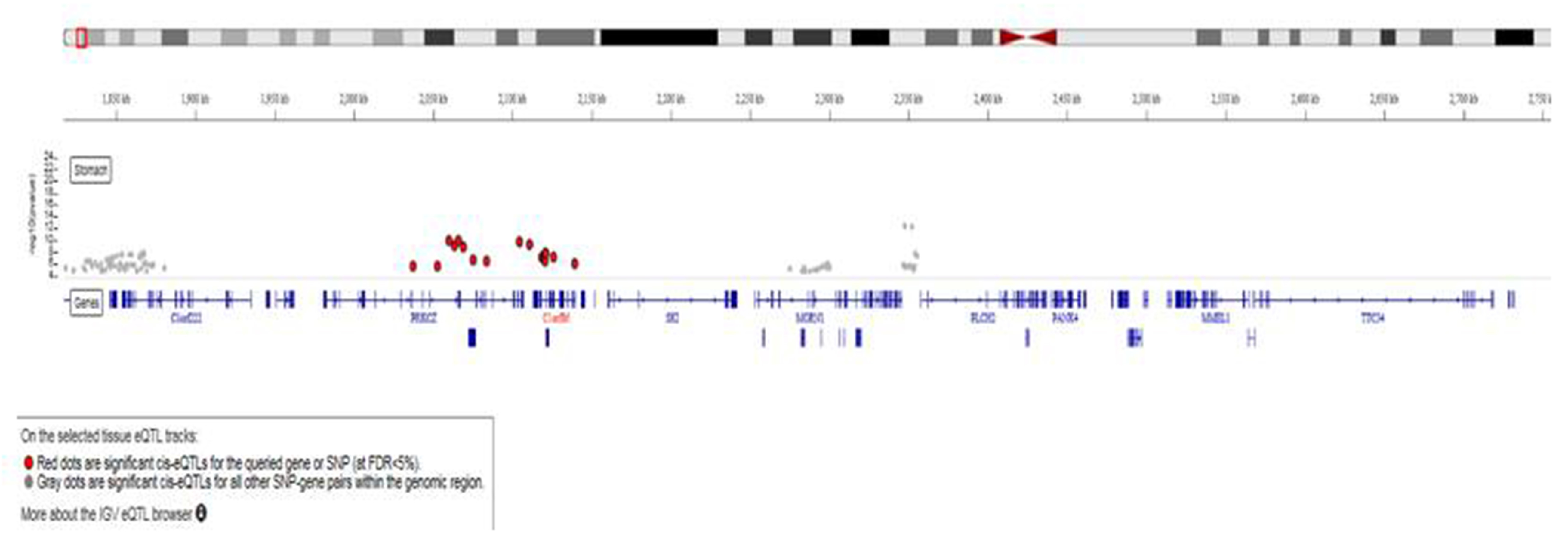

Supplement: S3 Fig — These genes (C1orf86, FAM213B, and HES5) from chromosome 1(Chr1:1819996–3028345) are copy gain genes. Colour codes with red-dots are significant cis-eQTLS for the queried gene which less than at FDR<0.5 and grey-dots are significant cis-eQTLS for all other SNP-genes within the genomic region. This was used for Broading software (http://software.broadinstitute.org/software/igv/home). (TIF) [file pone.0202576.s005.tif]

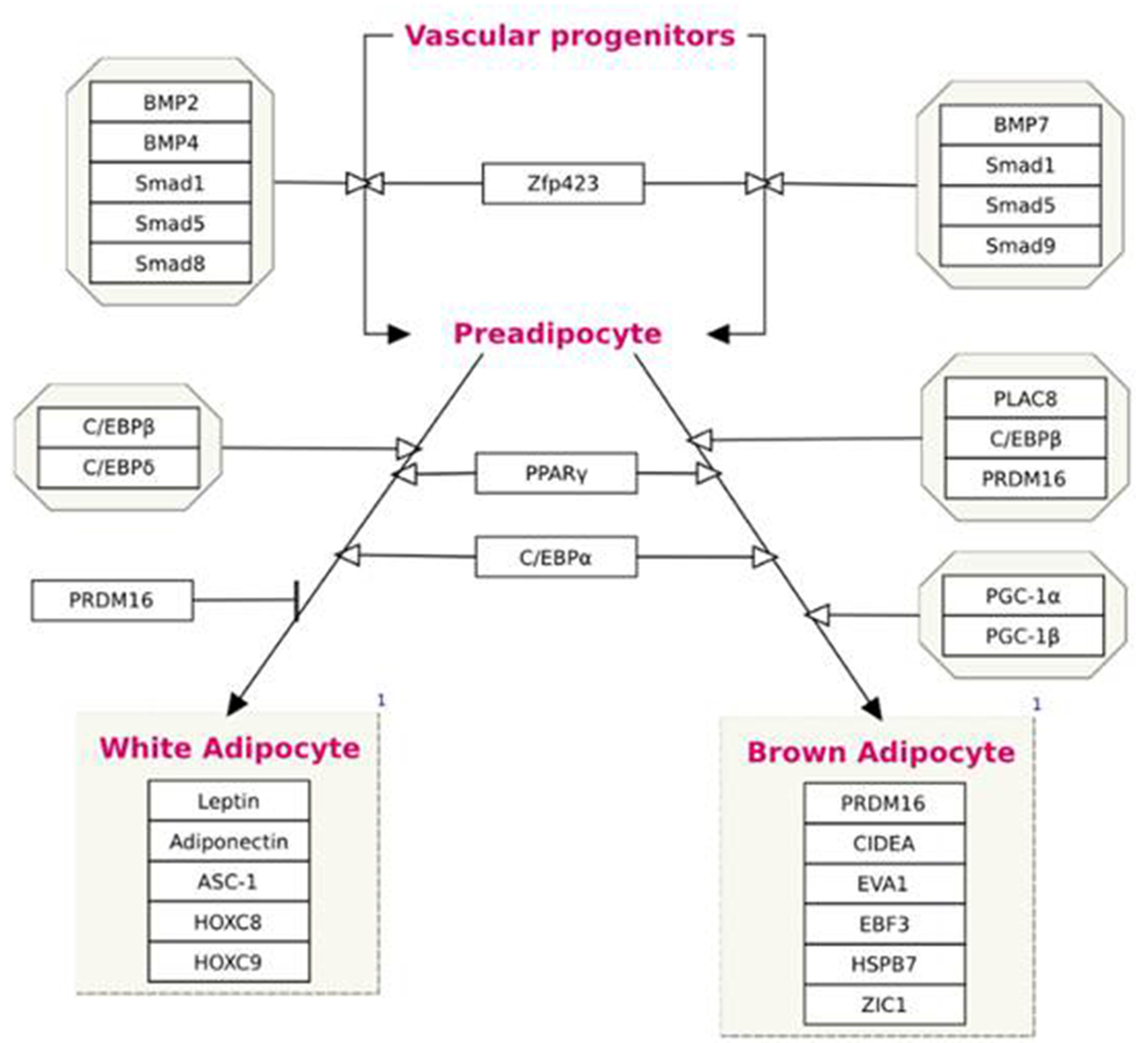

Supplement: S4 Fig — (TIF) [file pone.0202576.s006.tif]

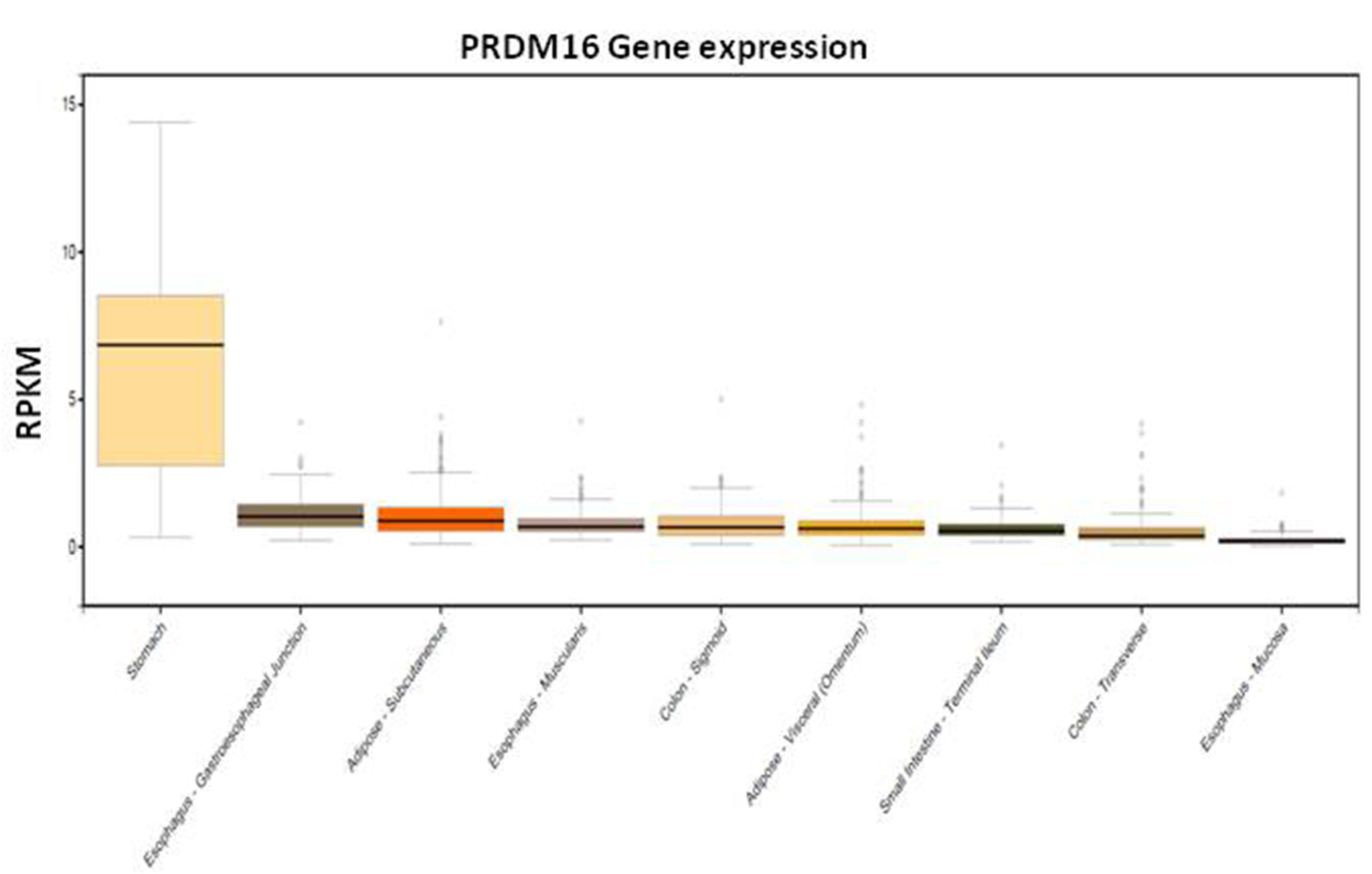

Supplement: S5 Fig — Median, the first and the third quartile were calculated. This was used for Broading software (http://software.broadinstitute.org/software/igv/home). (TIF) [file pone.0202576.s007.tif]

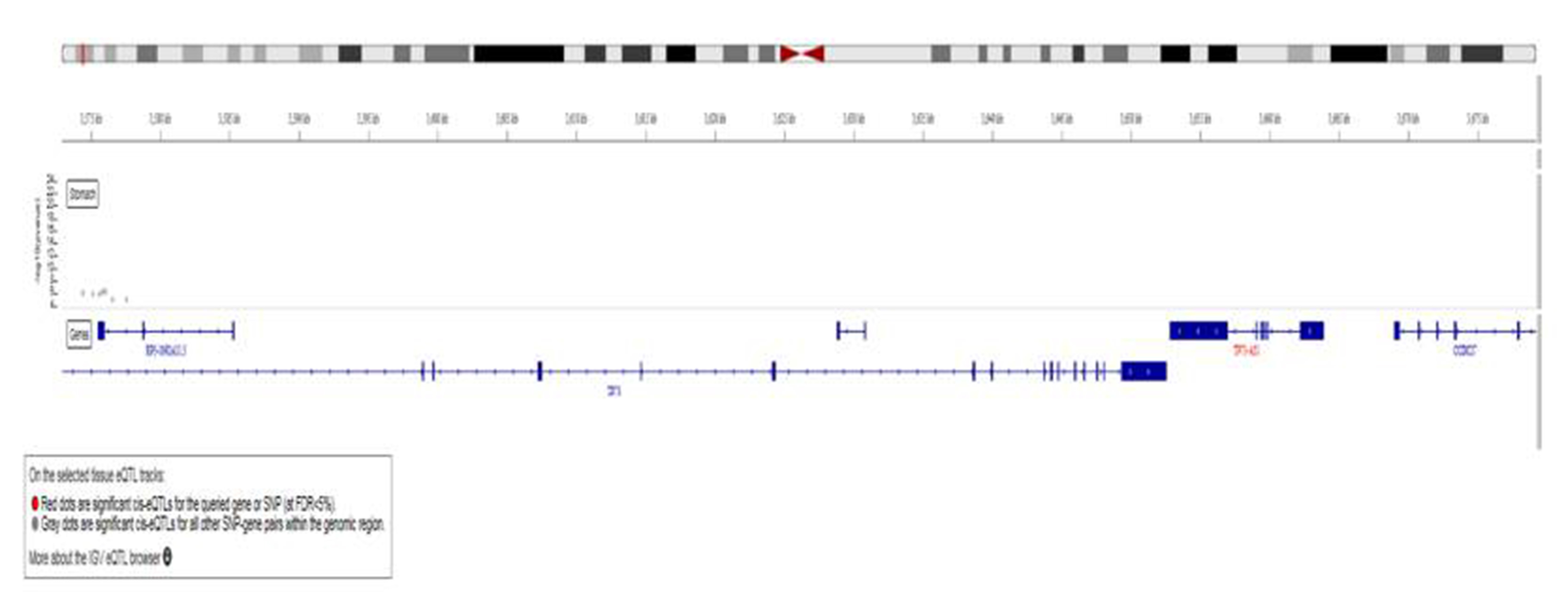

Supplement: S6 Fig — These genes (TP73 and TP73-AS1) from chromosome 1(Chr1:3572898–3679203) LOH region. Colour codes, with red-dots are significant cis-eQTLS for the queried gene which less than at FDR<0.5 and grey- dots are significant cis-eQTLS for all other SNP-genes within the genomic region.This was also used for Broading software (http://software.broadinstitute.org/software/igv/home). (TIF) [file pone.0202576.s008.tif]
